# Moment-to-moment interaction between affectivity and coping behaviours in bipolar disorder and the role of cognitive appraisals

**DOI:** 10.1192/bjo.2019.35

**Published:** 2019-05-23

**Authors:** Michelle Hoi-ting Leung, Suzanne Ho-wai So, Nate Tsz-Kit Kwok, Iris Hoi-ching Ng, Pui-shuen Chan, Chloe Chor-wing Lo, Shirley Na, Arthur Dun-ping Mak, Sing Lee

**Affiliations:** Clinical Psychology trainee, Department of Psychology, The Chinese University of Hong Kong, Hong Kong SAR, China; Associate Professor, Department of Psychology, The Chinese University of Hong Kong, Hong Kong SAR, China; Clinical Psychologist, Department of Psychology, The Chinese University of Hong Kong, Hong Kong SAR, China; Clinical Psychology trainee, Department of Psychology, The Chinese University of Hong Kong, Hong Kong SAR, China; Clinical Psychology trainee, Department of Psychology, The Chinese University of Hong Kong, Hong Kong SAR, China; Clinical Psychology trainee, Department of Psychology, The Chinese University of Hong Kong, Hong Kong SAR, China; Clinical Psychology trainee, Department of Psychology, The Chinese University of Hong Kong, Hong Kong SAR, China; Associate Professor, Department of Psychiatry, The Chinese University of Hong Kong, Hong Kong SAR, China; Professor, Department of Psychiatry, The Chinese University of Hong Kong, Hong Kong SAR, China

**Keywords:** Bipolar disorder, affect, coping, experience sampling, cognitive appraisals

## Abstract

**Background:**

Individuals with bipolar disorder respond to affective symptoms with a range of coping behaviours, which may further maintain the symptoms.

**Aims:**

To examine moment-to-moment dynamics between affective states and coping behaviours, and to evaluate the role of cognitive appraisals of internal states as moderators.

**Method:**

Forty-six individuals with bipolar disorder completed a clinical interview and an experience sampling assessment over 6 days. Time-lagged analyses were conducted by multilevel regression modelling.

**Results:**

A total of 1807 momentary entries were analysed. Negative affect predicted an increase in rumination at the subsequent time point (*β* = 0.21, s.e. = 0.08, *P* = 0.009, 95% CI 0.05–0.36), and *vice versa* (*β* = 0.03, s.e. = 0.01, *P* = 0.009, 95% CI 0.01–0.05). Positive affect predicted an increase in adaptive coping (*β* = 0.26, s.e. = 0.11, *P* = 0.018, 95% CI 0.04–0.47), and *vice versa* (*β* = 0.02, s.e. = 0.01, *P* = 0.019, 95% CI 0.00–0.03). Positive affect also predicted a decrease in rumination (*β* = −0.15, s.e. = 0.06, *P* = 0.014, 95% CI −0.26 to −0.03), and *vice versa* (*β* = −0.03, s.e. = 0.01, *P* = 0.016, 95% CI −0.06 to −0.01). Extreme cognitive appraisals predicted stronger associations between affective states and coping behaviours.

**Conclusions:**

Feedback loops between affective states and coping behaviours were revealed in the daily life of individuals with bipolar disorder, which were moderated by extreme cognitive appraisals.

**Declaration of interest:**

None.

Bipolar disorder is characterised by instability of affect within and between episodes. There has been an increase in interest in understanding how affective symptoms are maintained by an individual's behaviours. Researchers have suggested that individuals with bipolar disorder engage in coping behaviours in an attempt to control or regulate their symptoms, with maladaptive coping behaviours in turn exacerbating the affective symptoms.^1^

## Relationship between affective states and coping behaviours

The association between affective states and coping behaviour has recently been tested using experience sampling methodology (ESM). In Pavlickova *et al*,^2^ level of negative affect was found to predict an increase in rumination at the next assessment time point, whereas engagement in rumination predicted a subsequent increase in negative affect. On the contrary, positive affect was found to predict an increase in risk-taking behaviours, and risk-taking behaviours in turn increased positive affect. In two other experience sampling studies involving non-patient samples, Brans *et al*^3^ also reported that rumination was associated with increases in negative affect and decreases in positive affect. However, Pavlickova *et al*^2^'s finding of the association between positive affect and risk-taking behaviours has not been replicated. It is of note that in Pavlickova *et al*,^2^ the positive affect factor consisted of the following items: cheerful, excited, relaxed and satisfied. It is not clear whether these items represented elevated affect analogous to mania/hypomania (such as excessive excitement and arousal) or a pleasant and calm emotional state. Neurophysiological studies^4,5^ have suggested that elevated affect and positive affect are two distinct dimensions, with the former relating to drive and arousal that underpins activated and energised behaviours, and the latter relating to a sense of contentment, well-being and peacefulness when one is not actively seeking out resources. When positive affect consisted of being happy and relaxed (without the excitement and arousal component), Brans *et al*^3^ reported that adaptive coping strategies (such as reflection, distraction and social sharing) were associated with increases in positive affect. Therefore, it remains unclear whether positive affect (or its specific components) is more closely associated with adaptive coping or risk-taking. As elevated affect and positive affect are not positively correlated, but may be experienced simultaneously by individuals with bipolar disorder,^6^ understanding the relationship between affect and coping behaviour would be advanced if we assess positive affect and elevated affect separately as patients with bipolar disorder experience their daily lives. Therefore, the current study will test the moment-to-moment associations between a range of affective states (negative affect, positive affect and elevated affect) and coping behaviours (rumination, adaptive coping and risk-taking).

## Role of cognitive appraisals as moderator

Individuals with bipolar disorder have a tendency to appraise activated internal states as extremely positive or negative.^1^ It was suggested that positive appraisals of internal states predicted activation via engagement in ascent behaviour (such as risk-taking), and that negative appraisals of internal states predicted engagement in descent behaviour (such as rumination).^7^ However, the role of cognitive appraisals on moment-to-moment interactions between affective states and coping behaviour has not been directly tested.

## Aim of study

The present study aimed to examine the moment-to-moment associations between affective states and coping behaviour, and the effect of baseline level of extreme cognitive appraisals on these moment-to-moment relationships. Key hypotheses were as follows:

- Momentary level of affective states will drive changes in coping behaviour at the next assessment time point:
  - Level of negative affect at time *t* will predict an increase in rumination at time *t* + 1,
  - Level of positive affect at time *t* will predict an increase in adaptive coping at time *t* + 1,
  - Level of elevated affect at time *t* will predict an increase in risk taking at time *t* + 1;
- Momentary engagement in coping behaviour will drive changes in affective states at the next assessment time point:
  - Level of rumination at time *t* will predict an increase in negative affect at time *t* + 1,
  - Level of adaptive coping at time *t* will predict an increase in positive affect at time *t* + 1,
  - Level of risk taking at time *t* will predict an increase in elevated affect at time *t* + 1;
- Baseline level of extreme cognitive appraisals will moderate the moment-to-moment relationship between affective states and coping behaviours. Specifically, extreme cognition will predict stronger associations between negative affect and rumination, and between elevated affect and risk-taking.

## Method

### Sample

Ethical approval for the study was granted by the Joint Chinese University of Hong Kong and New Territories East Cluster Clinical Research Ethics Committee (CRE-2013-652-T). Inclusion criteria were: outpatients with a diagnosis of bipolar spectrum disorder, age 18 years or above and an ability to read Chinese and to complete self-report measures independently. Exclusion criteria were intellectual disability, brain injury and a primary diagnosis of substance-related disorder or organic bipolar disorder.

### Measures

#### ESM

ESM is a diary method that collects participants' self-reports at various moments throughout the day and has been applied in examining psychopathology among individuals with bipolar disorder.^2,8^ In our current study, moment-to-moment levels of affective symptoms and coping behaviours were assessed by an assessment app on an electronic device. The app (named ‘Questionnaire’) was designed and developed by the Clinical Psychology Laboratory of the Chinese University of Hong Kong, and has been used in other studies.^9^ The Questionnaire app can be downloaded through the iOS and Android platforms. Consistent with other ESM studies,^2,10,11^ our app generated ten moments each day, over 6 consecutive days. The ESM signal was emitted at quasi-random intervals during the day (10.00–22.00 h). Care was taken so that the ESM assessment captured participants' flow of daily life without disrupting their routine. Upon communication with individual patients, we made adjustment to the signalling schedule according to their daily routine and sleep hours, as appropriate.

With reference to previous ESM studies,^12,13^ three items were included for negative affect (irritated, low and tense) and positive affect (cheerful, relaxed and content), respectively. In addition, we included three new items to measure elevated affect (excited, elevated and energetic). These items were assessed on a seven-point Likert scale (from 1, ‘not at all’, to 7, ‘very much’). We reported acceptable to excellent internal consistency for each set of affective items (Cronbach's *α* = 0.82 for negative affect, 0.92 for positive affect, and 0.72 for elevated affect).

Based on a revised version of the Response Style Questionnaire,^14^ a total of ten ESM items (see Appendix) were included to assess participants' use of a range of coping strategies, including rumination, distraction, problem-solving and risk-taking. Following a previous study,^2^ ‘distraction’ and ‘problem-solving’ were grouped under ‘adaptive coping’. These three coping components (rumination, risk-taking and adaptive coping) yielded satisfactory levels of internal consistency in our study (Cronbach's *α* = 0.63–0.92).

#### Clinical interview

All participants completed a clinical assessment interview at baseline. Psychiatric diagnosis was determined using the Chinese-bilingual Structured Clinical Interview for DSM-IV (Axis I, Patient version).^15^ Depressive symptoms, manic symptoms and anxiety symptoms were assessed by the ten-item Montgomery–Asberg depression rating scale (MADRS),^16^ the 11-item Young Mania Rating Scale (YMRS)^17^ and the 14-item Hamilton Rating Scale for Anxiety (HRSA),^18^ respectively. The score range was 0–60 for the MADRS and YMRS, and 0–56 for the HRSA.

#### Cognitive appraisals of changes in internal states

The Hypomanic Attitudes and Positive Predictions Inventory (HAPPI) is a 26-item self-report inventory that was designed to measure extreme and personalised positive and negative appraisal style of internal states for individuals with bipolar disorder.^19^ It was validated in patients with bipolar disorder and nonclinical control samples.^1^ The HAPPI yielded five factor scores: catastrophic beliefs about internal states, reduced social regulation, an activating response style, success activation and triumph over fear, and loss of control when activated. HAPPI has been translated into Chinese for this study. Graduate-level students conducted the translation and back-translation procedures independently. Any discrepancy was resolved by discussion. The translated version of the HAPPI showed excellent internal consistencies in the current sample (Cronbach's *α* for the factor scores ranging from 0.91 to 0.97).

### Procedure

Participants were either referred by clinicians or volunteered to take part in this study. Their suitability for study participation was confirmed by their psychiatrists. All participants provided written consent before the start of the study. At the end of the baseline interview, participants completed a set of questionnaires and were guided through the use of the ESM app individually. Over the following 6 days, participants completed the ESM on their own mobile phone or on an iPod Touch borrowed from our laboratory. To increase compliance to the assessment, participants were contacted at least twice during the 6-day period; those who had difficulty in using the app received further support and training.

### Statistical analysis

Statistical analysis was carried out using hierarchical linear modelling, a statistical approach that accounts for within person (level 1) and between-person (level 2) variance. Multilevel regression modelling with maximum likelihood estimation was used to model the relationships between momentary variables, with the assumption that data are missing at random.^20^ To model between-moment associations within the same day, new variables for each lagged level-1 variable at its subsequent moment was named *t* *+* *1*. We modelled the effect of the level-1 independent variable (IV) on change in level-1 dependent variable (DV) by testing the regression of DV_*t+1*_ on IV_*t*_, controlling for DV at moment *t* (DV_*t*_). Following Delespaul's^21^ guideline as well as previous ESM studies,^2,9–11^ participants who completed less than one-third of total entries (i.e. 20 valid reports) were excluded from the analysis.

To examine whether any of the identified main effects were moderated by patients' diagnostic group or episodic status (i.e. their level-2 clinical characteristics), we conducted three sensitivity analyses. For each of the identified main effects, an interaction term between the significant IV and patient's diagnostic group (bipolar type 1 versus type 2), an interaction term between the significant IV and patient's episodic status (in an active episode versus not in an active episode) and an interaction term between the significant IV and gender (male versus female) were tested in additional regression models.

To test the role of extreme cognition on the moment-to-moment main effects, we modelled the interaction effects of extreme cognition as level-2 IV and affective states as level-1 IV_*t*_ on coping behaviour as DV_*t+1*_, controlling for DV_*t*_. Data analysis was conducted on Stata version 12 for Windows. Statistical significance was set at *P* < 0.05.

## Results

### Demographic and clinical data

Among 125 referrals, 64 fulfilled the inclusion criteria, among whom 46 completed at least 20 ESM entries and were included in data analysis, which is comparable with other recent ESM studies.^2,9,11^ In total, the participants completed 1807 ESM entries, with a mean completion rate of 65.85% (range 35–100%), which was calculated as the number of completed entries divided by the total number of entries expected over the 6-day period.

As shown in Table 1, among the 46 participants, 11 (23.90%) were males and 35 (76.10%) were females. Average age was 38.93 years (range 18–61). Participants had received psychiatric service for an average of 10.77 years (range 0–40), with a majority of the sample (*n* = 43, 93.48%) being on psychiatric medication. Thirty-three individuals (71.73%) had a diagnosis of bipolar type 1 disorder and 13 (28.26%) had a diagnosis of bipolar type 2 disorder. The numbers of participants who met the DSM-IV criteria for a depressive episode, hypomanic/manic episode or and mixed episode in the past month were five (10.87%), seven (15.22%) and seven (15.22%), respectively.

**Table 1:** Demographic information of the sample (*N* = 46)

Characteristics	*N* (%)	Mean (s.d.)	Median	q25 and q75
Age (years)		38.93 (12.01)	39	31, 49
Gender				
Male	11 (23.90%) 35 (76.10%)			
Female				
Education level		13.07 (3.23)	13	11, 16
Bipolar disorder diagnosis				
Bipolar type 1 disorder	33 (71.73%)			
Bipolar type 2 disorder	13 (28.26%)			
Onset of bipolar disorder		10.77 (10.71)	8.5	2, 16
>3 years	16 (36.36%)			
≤3 years	28 (63.64%)			
Number of admissions		2.09 (2.71)	1	0, 2
Bipolar episode in the past month				
Not in an active episode	27 (58.70%)			
Depressive episode	5 (10.87%)			
Hypomanic/manic episode	7 (15.22%)			
Mixed episode (depressive and hypomanic/manic)	7 (15.22%)			
Baseline mood symptoms				
Young Mania Rating Scale		2.16 (3.90)	0	0, 2
Montgomery–Asberg Depression Rating Scale		6.66 (7.97)	2.5	0, 12.5
Hamilton Rating Scale for Anxiety		9.32 (8.77)	6.5	2, 16

Baseline mood symptom severity scores were as follows: YMRS = 2.16 (s.d. = 3.90), MADRS = 6.66 (s.d. = 7.97) and HRSA = 9.32 (s.d. = 8.77). On MADRS,^16^ 26 participants (60.47%) scored within a normal range (0–6), 13 (30.23%) scored within a mild range (7–19) and four (9.30%) sored within a moderate range (20–34). On HRSA,^18^ 34 participants (79.07%) scored within a mild range (0–17), six (13.95%) scored within a mild-to-moderate range (18–24) and three (6.98%) scored within a moderate range (25–30).

Average affect scores across 6 days, as assessed by ESM (score range 1–7), were as follows: negative affect, 2.37 (s.d. = 1.39); positive affect, 4.08 (s.d. = 1.74) and elevated affect, 1.82 (s.d. = 1.08). For participants who were not currently in an active episode (*n* = 27), average affect scores across 6 days were as follows: negative affect, 2.01 (s.d. = 1.20); positive affect, 4.48 (s.d. = 1.71) and elevated affect, 1.90 (s.d. = 1.13). Average coping scores across 6 days, as assessed by ESM (score range 1–7), were as follows: rumination, 2.35 (s.d. = 1.62); risk-taking 1.30 (s.d. = 0.78) and adaptive coping 3.16 (s.d. = 1.82).

### Between-moment prediction from affective states to coping behaviours

Moment-to-moment relationships between affective states at time *t* and coping behaviours at time *t* *+* *1* are shown in Table 2. After controlling for rumination_*t*_, level of negative affect_*t*_ predicted an increase in rumination_*t+1*_ (model 2: *β* = 0.21, s.e. = 0.08, *P* = 0.009, 95% CI 0.05–0.36) and level of positive affect_*t*_ predicted a decrease in rumination_*t+1*_ (model 5: *β* = −0.15, s.e. = 0.06, *P* = 0.014, 95% CI −0.26 to −0.03). After controlling for risk-taking_*t*_, level of negative affect_*t*_ predicted an increase in risk taking_*t+1*_ (model 1: *β* = 0.07, s.e. = 0.03, *P* = 0.028, 95% CI 0.01–0.14). After controlling for adaptive coping_*t*_, level of positive affect_*t*_ predicted an increase in adaptive coping_*t+1*_ (model 6: *β* = 0.26, s.e. = 0.11, *P* = 0.018, 95% CI 0.04–0.47).

**Table 2:** Between-moment regression of coping behaviours on affective states (models 1–9) and between-moment regression of affective states on coping behaviours (models 10–18)

Model	Independent variable	Dependent variable	*β* (s.e.)	95% CI	*P* value
1	**Negative affect_*t*_**	Risk-taking_*t+1*_	0.07 (0.03)	0.01–0.14	0.028
	Risk-taking_*t*_		0.24 (0.03)	0.19–0.30	<0.001
2	**Negative affect_*t*_**	Rumination_*t+1*_	0.21 (0.08)	0.05–0.36	0.009
	Rumination_*t*_		0.22 (0.03)	0.16–0.28	<0.001
3	**Negative affect_*t*_**	Adaptive coping_*t+1*_	−0.10 (0.14)	−0.39–0.18	0.473
	Adaptive coping_*t*_		0.20 (0.03)	0.14–0.26	<0.001
4	**Positive affect_*t*_**	Risk-taking_*t+1*_	−0.03 (0.03)	−0.08–0.02	0.174
	Risk-taking_*t*_		0.25 (0.03)	0.19–0.30	<0.001
5	**Positive affect_*t*_**	Rumination_*t+1*_	−0.15 (0.06)	−0.26–−0.03	0.014
	Rumination_*t*_		0.22 (0.03)	0.16–0.28	<0.001
6	**Positive affect_*t*_**	Adaptive coping_*t+1*_	0.26 (0.11)	0.04–0.47	0.018
	Adaptive coping_*t*_		0.19 (0.03)	0.13–0.25	<0.001
7	**Elevated affect_*t*_**	Risk-taking_*t+1*_	−0.07 (0.04)	−0.15–0.01	0.108
	Risk-taking_*t*_		0.25 (0.03)	0.19–0.30	<0.001
8	**Elevated affect_*t*_**	Rumination_*t+1*_	−0.00 (0.10)	−0.20–0.19	0.974
	Rumination_*t*_		0.23 (0.03)	0.17–0.29	<0.001
9	**Elevated affect_*t*_**	Adaptive coping_*t+1*_	0.34 (0.18)	−0.01–0.70	0.058
	Adaptive coping_*t*_		0.19 (0.03)	0.13–0.25	<0.001
10	**Risk-taking_*t*_**	Negative affect_*t+1*_	0.02 (0.02)	−0.03–0.07	0.414
	Negative affect_*t*_		0.21 (0.03)	0.16–0.27	<0.001
11	**Risk-taking_*t*_**	Positive affect_*t+1*_	0.04 (0.03)	−0.02–0.10	0.234
	Positive affect_*t*_		0.18 (0.03)	0.12–0.23	<0.001
12	**Risk-taking_*t*_**	Elevated affect_*t+1*_	0.01 (0.02)	−0.03–0.04	0.748
	Elevated affect_*t*_		0.26 (0.03)	0.21–0.32	<0.001
13	**Rumination_*t*_**	Negative affect_*t+1*_	0.03 (0.01)	0.01–0.05	0.009
	Negative affect_*t*_		0.20 (0.03)	0.15–0.26	<0.001
14	**Rumination_*t*_**	Positive affect_*t+1*_	−0.03 (0.01)	−0.06–−0.01	0.016
	Positive affect_*t*_		0.17 (0.03)	0.12–0.22	<0.001
15	**Rumination_*t*_**	Elevated affect_*t+1*_	0.00 (0.01)	−0.01–0.02	0.843
	Elevated affect_*t*_		0.26 (0.03)	0.21–0.32	<0.001
16	**Adaptive coping_*t*_**	Negative affect_*t+1*_	−0.01 (0.01)	−0.02–0.01	0.319
	Negative affect_*t*_		0.21 (0.03)	0.16–0.27	<0.001
17	**Adaptive coping_*t*_**	Positive affect_*t+1*_	0.02 (0.01)	0.00–0.03	0.019
	Positive affect_*t*_		0.17 (0.03)	0.12–0.23	<0.001
18	**Adaptive coping_*t*_**	Elevated affect_*t+1*_	0.02 (0.00)	0.01–0.02	<0.001
	Elevated affect_*t*_		0.26 (0.03)	0.20–0.31	<0.001

The predictor variables that directly address hypotheses 2–3 are shown in bold.

Sensitivity analysis of diagnostic group revealed that the effect of positive affect_*t*_ on decrease in rumination_*t*+1_ was smaller in the bipolar type 2 group than the bipolar type 1 group (*β* = −0.30, s.e. = 0.14, *P* = 0.034, 95% CI −0.58 to −0.02). The other main effects from affective states to coping behaviours did not significantly differ across other diagnostic groups, episodic status or gender (all *P* > 0.05).

### Between-moment prediction from coping behaviours to affective states

Moment-to-moment relationships between coping behaviours at time *t* and affective states at time *t* *+* *1* are shown in Table 2. After controlling for negative affect_*t*_, level of rumination_*t*_ predicted an increase in negative affect_*t+1*_ (model 13: *β* = 0.03, s.e. = 0.01, *P* = 0.009, 95% CI 0.01–0.05). After controlling for positive affect_*t*_, level of rumination_*t*_ predicted a decrease in positive affect_*t+1*_ (model 14: *β* = −0.03, s.e. = 0.01, *P* = 0.016, 95% CI −0.06 to −0.01), whereas level of adaptive coping_*t*_ predicted an increase in positive affect_*t+1*_ (model 17: *β* = 0.02, s.e. = 0.01, *P* = 0.019, 95% CI 0.00–0.03). After controlling for elevated affect_*t*_, level of adaptive coping_*t*_ predicted an increase in elevated affect_*t+1*_ (model 18: *β* = 0.02, s.e. = 0.00, *P* < 0.001, 95% CI 0.01–0.02).

Sensitivity analysis of episodic status revealed that the effect of rumination_*t*_ on decrease in positive affect_*t+1*_ (*β* = −0.07, s.e. = 0.02, *P* < 0.001, 95% CI −0.11 to −0.03) and the effect of adaptive coping_*t*_ on increase in elevated affect_*t+1*_ (*β* = −0.03, s.e. = 0.01, *P* < 0.001, 95% CI −0.04 to −0.02) were weaker in individuals who were in an active episode than individuals who were not in active episode. The other main effects from coping behaviours to affective states did not significantly differ across diagnostic groups, other episodic status or gender (all *P* > 0.05).

**Fig. 1:**
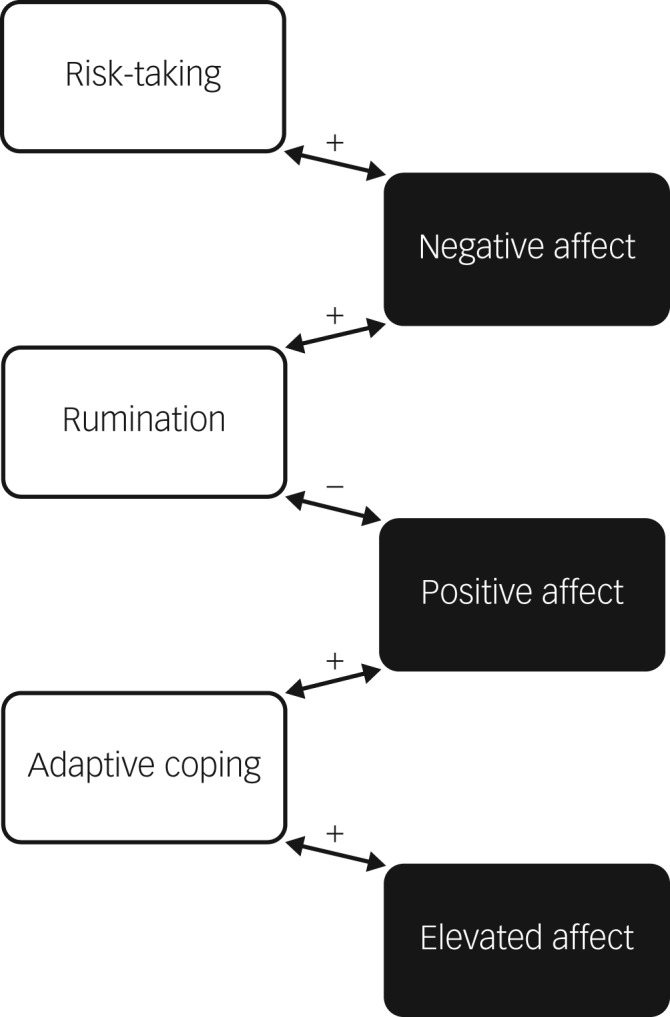
Schematic diagram illustrating time-lagged relationships between affective states and coping behaviours.

Figure 1 summarises the main effects of cross-moment relationships between affective states and coping behaviours in a schematic manner.

### Role of baseline level of extreme cognitive appraisals on the moment-to-moment relationship between affective states and coping behaviours

As shown in Table 3, time-lagged analysis demonstrated that all five factor scores of HAPPI predicted a stronger increase in rumination_*t+1*_ following negative affect_*t*_ (models 2a–2e), whereas all factors except factor IV of HAPPI predicted a more marked decrease in rumination_*t+1*_ following positive affect_*t*_ (models 5a–5d). All five factors of HAPPI predicted a stronger increase in adaptive coping_*t+1*_ following positive affect_*t*_ (models 6a–6e). Three factors predicted a stronger increase in risk taking_*t+1*_ following negative affect_*t*_ (models 1a–1c).

**Table 3:** Moderation effects of cognitive appraisal styles on the moment-to-moment relationships from affective states to coping behaviours

Model	Independent variable	Dependent variable	*β* (s.e.)	95% CI	*P* value
1a	Negative affect_*t*_	Risk-taking_*t+1*_	0.07 (0.03)	0.01–0.14	0.028
	Risk-taking_*t*_		0.24 (0.03)	0.19–0.30	<0.001
	**HAPPI factor 1 × negative affect_*t*_**		0.00 (0.00)	−0.00–0.00	0.049
	HAPPI factor 1 × risk-taking_*t*_		0.24 (0.03)	0.18–0.30	<0.001
1b	Negative affect_*t*_	Risk-taking_*t+1*_	0.07 (0.03)	0.01–0.14	0.028
	Risk-taking_*t*_		0.24 (0.03)	0.19–0.30	<0.001
	**HAPPI factor 3** **×** **negative affect_*t*_**		0.00 (0.00)	0.00–0.00	0.027
	HAPPI factor 3 × risk-taking_*t*_		0.24 (0.03)	0.19–0.30	<0.001
1c	Negative affect_*t*_	Risk-taking_*t+1*_	0.07 (0.03)	0.01–0.14	0.028
	Risk-taking_*t*_		0.24 (0.03)	0.19–0.30	<0.001
	**HAPPI factor 4** **×** **negative affect_*t*_**		0.00 (0.00)	0.00–0.00	0.038
	HAPPI factor 4 × risk-taking_*t*_		0.24 (0.03)	0.20–0.30	<0.001
2a	Negative affect_*t*_	Rumination_*t+1*_	0.21 (0.08)	0.05–0.36	0.009
	Rumination_*t*_		0.22 (0.03)	0.16–0.28	<0.001
	**HAPPI factor 1** **×** **negative affect_*t*_**		0.00 (0.00)	0.00–0.01	0.003
	HAPPI factor 1 × rumination_*t*_		0.22 (0.03)	0.16–0.28	<0.001
2b	Negative affect_*t*_	Rumination_*t+1*_	0.21 (0.08)	0.05–0.36	0.009
	Rumination_*t*_		0.22 (0.03)	0.16–0.28	<0.001
	**HAPPI factor 2** **×** **negative affect_*t*_**		0.01 (0.00)	0.00–0.01	0.002
	HAPPI factor 2 × rumination_*t*_		0.22(0.03)	0.16–0.28	<0.001
2c	Negative affect_*t*_	Rumination_*t+1*_	0.21 (0.08)	0.05–0.36	0.009
	Rumination_*t*_		0.22 (0.03)	0.16–0.28	<0.001
	**HAPPI factor 3** **×** **negative affect_*t*_**		0.00 (0.00)	0.00–0.01	0.014
	HAPPI factor 3 × rumination_*t*_		0.22 (0.03)	0.16–0.28	<0.001
2d	Negative affect_*t*_	Rumination_*t+1*_	0.21 (0.08)	0.05–0.36	0.009
	Rumination_*t*_		0.22 (0.03)	0.16–0.28	<0.001
	**HAPPI factor 4** **×** **negative affect_*t*_**		0.00 (0.00)	0.00–0.01	0.019
	HAPPI factor 4 × rumination_*t*_		0.22 (0.03)	0.16–0.28	<0.001
2e	Negative affect_*t*_	Rumination_*t+1*_	0.21 (0.08)	0.05–0.36	0.009
	Rumination_*t*_		0.22 (0.03)	0.16–0.28	<0.001
	**HAPPI factor 5** **×** **negative affect_*t*_**		0.00 (0.00)	0.00–0.01	0.030
	HAPPI factor 5 × rumination_*t*_		0.22 (0.03)	0.16–0.28	<0.001
5a	Positive affect_*t*_	Rumination_*t+1*_	−0.15 (0.06)	−0.26–−0.03	0.014
	Rumination_*t*_		0.22 (0.03)	0.16–0.28	<0.001
	**HAPPI factor 1** **×** **positive affect_*t*_**		−0.00 (0.00)	−0.01–0.00	0.001
	HAPPI factor 1 × rumination_*t*_		0.23 (0.03)	0.17–0.29	<0.001
5b	Positive affect_*t*_	Rumination_*t+1*_	−0.15 (0.06)	−0.26–−0.03	0.014
	Rumination_*t*_		0.22 (0.03)	0.16–0.28	<0.001
	**HAPPI factor 2** **×** **positive affect_*t*_**		−0.01 (0.00)	−0.01–0.00	<0.001
	HAPPI factor 2 × rumination_*t*_		0.22 (0.03)	0.16–0.28	<0.001
5c	Positive affect_*t*_	Rumination_*t+1*_	−0.15 (0.06)	−0.26–−0.03	0.014
	Rumination_*t*_		0.22 (0.03)	0.16–0.28	<0.001
	**HAPPI factor 3** **×** **positive affect_*t*_**		−0.00 (0.00)	−0.01–0.00	0.029
	HAPPI factor 3 × rumination_*t*_		0.22 (0.03)	0.16–0.28	<0.001
5d	Positive affect_*t*_	Rumination_*t+1*_	−0.15 (0.06)	−0.26–−0.03	0.014
	Rumination_*t*_		0.22 (0.03)	0.16–0.28	<0.001
	**HAPPI factor 5** **×** **positive affect_*t*_**		−0.00 (0.00)	−0.01–0.00	0.009
	HAPPI factor 5 × rumination_*t*_		0.22 (0.03)	0.16–0.28	<0.001
6a	Positive affect_*t*_	Adaptive coping_*t+1*_	0.26 (0.11)	0.04–0.47	0.018
	Adaptive coping_*t*_		0.19 (0.03)	0.13–0.25	<0.001
	**HAPPI factor 1** **×** **positive affect_*t*_**		0.01 (0.00)	0.01–0.01	0.002
	HAPPI factor 1 × adaptive coping_*t*_		0.20 (0.03)	0.14–0.26	<0.001
6b	Positive affect_*t*_	Adaptive coping_*t+1*_	0.26 (0.11)	0.04–0.47	0.018
	Adaptive coping_*t*_		0.19 (0.03)	0.13–0.25	<0.001
	**HAPPI factor 2** **×** **positive affect_*t*_**		0.01 (0.00)	0.01–0.01	0.009
	HAPPI factor 2 × adaptive coping_*t*_		0.20 (0.03)	0.14–0.26	<0.001
6c	Positive affect_*t*_	Adaptive coping_*t+1*_	0.26 (0.11)	0.04–0.47	0.018
	Adaptive coping_*t*_		0.19 (0.03)	0.13–0.25	<0.001
	**HAPPI factor 3** **×** **positive affect_*t*_**		0.01 (0.00)	0.00–0.01	0.008
	HAPPI factor 3 × adaptive coping_*t*_		0.20 (0.03)	0.14–0.26	<0.001
6d	Positive affect_*t*_	Adaptive coping_*t+1*_	0.26 (0.11)	0.04–0.47	0.018
	Adaptive coping_*t*_		0.19 (0.03)	0.13–0.25	<0.001
	**HAPPI factor 4** **×** **positive affect_*t*_**		0.01 (0.00)	0.00–0.01	0.025
	HAPPI factor 4 × adaptive coping_*t*_		0.20 (0.03)	0.14–0.26	<0.001
6e	Positive affect_*t*_	Adaptive coping_*t+1*_	0.26 (0.11)	0.04–0.47	0.018
	Adaptive coping_*t*_		0.19 (0.03)	0.13–0.25	<0.001
	**HAPPI factor 5** **×** **positive affect_*t*_**		0.01 (0.00)	0.00–0.01	0.042
	HAPPI factor 5 × adaptive coping_*t*_		0.19 (0.03)	0.13–0.25	<0.001

Only models with a significant Hypomanic Attitudes and Positive Predictions Inventory (HAPPI) factor × independent variable interaction term are included in this table. The predictor variables that directly address hypothesis 3 are shown in bold. HAPPI factor 1, catastrophic beliefs about internal states; HAPPI factor 2, reduced social regulation; HAPPI factor 3, activating response style; HAPPI factor 4, success activation and triumph over fear; HAPPI factor 5, loss of control when activated.

## Discussion

In this study, we modelled moment-to-moment interaction between various affective states and coping behaviours and tested the role of cognitive appraisals on these moment-to-moment dynamics among 46 individuals with bipolar disorder. By using a fine-grained experience sampling assessment, we delineated how one variable drives subsequent changes in another variable and captured the intricate relationships between affect and behaviour in a naturalistic manner. Major findings are as follows: (a) negative affect predicted an increase in rumination in the next assessment time point, and *vice versa*; (b) positive affect predicted a decrease in rumination and an increase in adaptive coping in the next assessment time point, and *vice versa* and (c) extreme cognitive appraisals predicted stronger associations between positive and negative affective states and coping behaviours.

Coping behaviours are carried out in an attempt to control or regulate affective symptoms.^1^ Although ascent behaviours (such as risk-taking and alcohol use) contribute to increases in activation levels, descent behaviours (such as rumination and social withdrawal) contribute to decreases in activation levels. In the current study, we found a feedback loop between rumination and affective states, whereby individuals ruminated more after experiencing negative affect and less after experiencing positive affect. The vicious cycle became self-perpetuating as rumination in turn led to more negative affect and less positive affect. Our results, together with Pavlickova *et al*^2^ and the depression literature, suggest that rumination maintains negative emotions in bipolar disorder in a similar way as in unipolar depression. As rumination contributes to maintenance of disruptive affective disturbance in individuals with bipolar disorder, our result raises the possibility of using rumination-based interventions, which have proven efficacy in major depressive disorder,^22^ for treating emotion disturbances in bipolar disorder.

We included separate ESM items for assessing elevated affect and positive affect. Rather than combining these items into one factor as in Pavlickova *et al*,^2^ our measures allowed us to differentiate the specific relationship between coping behaviours with an elevated (hypo)manic state as opposed to a relaxed/content state. When assessing elevated affect and positive affect separately, the association between risk-taking and positive affect reported by Pavlickova *et al*^2^ was no longer present. Instead, our results revealed that positive affect (i.e. a relaxed/content state) predicted subsequent engagement in adaptive coping. The positive feedback loop between positive affect and adaptive coping suggests that engaging in adaptive coping rather than rumination is helpful for eliciting positive affective experiences in individuals with bipolar disorder.

Contrary to our hypothesis, risk-taking did not predict any affective states. The lack of association may be partly explained by a generally low level of risk-taking in our sample (mean ESM score, 1.30). Although negative affect predicted risk-taking, we take caution in interpreting this finding as the effect was small and preliminary.

Our sensitivity analyses revealed that none of the eight cross-moment associations between affective states and coping behaviours differed across genders. Only one cross-moment association significantly differed between diagnostic group (i.e. the effect of positive affect_*t*_ on decrease in rumination_*t+1*_ was smaller in the bipolar type 2 group than the bipolar type 1 group), and two differed between patients who were versus were not in an active episode (i.e. the effect of rumination_*t*_ on decrease in positive affect _*t+1*_ and the effect of adaptive coping_*t*_ on increase in elevated affect _*t+1*_ were weaker among individuals who were in an active episode than individuals who were not in active episode). Therefore, the moment-to-moment relationships between affective states and coping behaviours were relatively robust and were not totally explained by diagnosis or clinical status. On speculation of the regression coefficients, it is tempting to argue that effects from affective states to coping behaviours are stronger than the effects from coping behaviours to affective states. However, as the overall effect sizes are small, this claim cannot be conclusive until our findings are replicated.

Extreme appraisals of internal states catastrophise the meaning of internal states, leading to engagement in maladaptive coping behaviours among individuals with bipolar disorder.^1^ This theoretical claim was partially supported by our data. For example, individuals who scored high on ‘success activation and triumph over fear’ (factor IV) tended to cope with negative affect by seeking sensation (e.g. alcohol use and reckless behaviours). This might lend support to the argument that risk-taking behaviour is adopted to restore individuals' sense of self and to lower their level of anxiety and depression.^23^ However, since cognitive appraisals exacerbated the positive cycle between positive affect and adaptive coping as well, it is also possible that individuals with more extreme cognitive appraisals are more likely to cope with their affective states in general, regardless of emotional valence.

### Limitations

There were several limitations to our study. First, although our sample was representative of individuals with bipolar disorder in an out-patient setting, it is hard to evaluate to what extent heterogeneity in clinical characteristics (including duration of illness, medication regime and comorbidity etc.) would have affected the results. Second, the current study was also limited by a lack of a control group. A healthy control group could help us discern the extent to which the association between affective states and coping behaviours is specific to the bipolar disorder group. A psychiatric control group that had heightened emotionality (e.g. pathological gamblers, or individuals with major depressive disorder or borderline personality disorder) would also be a welcomed addition to this area of research. Third, as the ESM signal scheduling was generated by a computer program in a pseudo-random manner, the duration of time-lag varied between days and persons. Therefore, we could not conclude how long it took for the moment-to-moment associations to occur. Fourth, our ESM completion rate was 65%, which was relatively low when compared with previous studies. Finally, we considered endorsement of the Response Style Questionnaire^14^ items as representing behavioural coping. It is possible that some of the items might happen infrequently or are formulated in a rather extreme way, hence explaining the relatively low endorsement rate. More importantly, unless we explicitly ask the individuals for the reason of their behaviours, it remained an assumption that individuals engage in behaviours such as risk-taking or rumination so as to cope with their affective states. As newer coping strategies such as acceptance and tolerance under the mindfulness conceptualisation have gained increased attention in the field and have been found to be effective in reducing symptom severity,^24^ additional coping strategies could be included in future research.

### Clinical implications

ESM supplements traditional assessment methods, and is particularly well suited for assessing subjective experiences of a fluctuating nature. These individually generated ESM data may offer potentially useful information for psychoeducation and intervention. As has been shown in treatment for depression,^25^ associations between emotional states and behaviour as captured by ESM can be discussed between the patient and therapist in the context of functional analysis and coping enhancement within the cognitive behavioural therapy framework. Moreover, if the effect of extreme cognitive appraisals on affective symptoms and coping behaviours is replicated, this could shed light on targeting cognitive appraisals as a potential therapeutic mechanism for psychological treatment for bipolar disorder.

## Appendix

**Appendix:** Experience sampling methodology items on engagement in coping behaviour (based on Nolen-Hoeksema's Response Style Questionnaire^26^

‘Between this beep and the previous beep, how much have you engaged in the following behaviour?’	
1. Make a plan to overcome a problem	1 (not at all) – 7 (very much)
2. Talk it out with someone whose opinions you respect (friend, clergy, family, etc.)	1 (not at all) – 7 (very much)
3. Seek out and engage in casual sexual relations	1 (not at all) – 7 (very much)
4. Think about how sad you feel	1 (not at all) – 7 (very much)
5. Do something enjoyable	1 (not at all) – 7 (very much)
6. Drink alcohol excessively	1 (not at all) – 7 (very much)
7. Think about how alone you feel	1 (not at all) – 7 (very much)
8. Think ‘I'm going to do something to make myself feel better’	1 (not at all) – 7 (very much)
9. Isolate yourself and think about the reasons why you feel sad	1 (not at all) – 7 (very much)
10. Do something reckless or dangerous	1 (not at all) – 7 (very much)

Items 1, 2, 5 and 8 represent adaptive coping; items 3, 6 and 10 represent risk-taking; and items 4, 7 and 9 represent rumination.
